# Factors Associated with Caesarean and Peripartum Complications at Southern Mozambique’s Rural Hospitals: A Cross-Sectional Analytical Study

**DOI:** 10.3390/healthcare10061013

**Published:** 2022-05-31

**Authors:** Sérgio Chicumbe, Group POEM, Maria do Rosário Oliveira Martins

**Affiliations:** 1 Health System and Policy Program, Instituto Nacional de Saúde, Estrada Nacional 1, Vila de Marracuene CEP 0205-02, Maputo 1120, Mozambique; muiangac@who.int; 2 Group for Performance in Obstetrics and Evaluation of Maternities (POEM), Instituto Nacional de Saúde, Estrada Nacional 1, Vila de Marracuene CEP 0205-02, Maputo 1120, Mozambique; 3 Global Health and Tropical Medicine, Instituto de Higiene e Medicina Tropical, Universidade NOVA de Lisboa, Rua da Junqueira No. 100, 1349-008 Lisbon, Portugal; mrfom@ihmt.unl.pt

**Keywords:** factors, caesarean, peripartum complications, rural, Mozambique

## Abstract

Information about factors underlying peripartum complications is needed to inform health programs in Mozambique. This retrospective study covered the period from January 2013 to December 2018 and was performed at three rural-district hospitals in southern Mozambique, aiming at assessing factors associated with caesarean and peripartum complications. Data were extracted by clinical criteria-based audits on randomly select clients’ files. Logistical regression was used to identify factors associated with peripartum complications. Amongst 5068 audited files, women mean age was 25 years (Standard Deviation (SD) = 7), gestational age was 38 weeks (SD = 2), 25% had “high obstetric-risk” and 19% delivered by caesarean. Factors significantly associated with caesarean included being transferred [Adjusted Odds Ratio (aOR) =1.8; 95% Confidence Interval (95%CI) = 1.3–2.6], preeclampsia [aOR (95%CI) = 2.0 (1.2–3.3)], age [aOR (95%CI) = 0.96 (0.93–0.99)] and “high obstetric-risk” [aOR (95%CI) = 0.54 (0.37–0.78)]. Factors significantly associated with neonatal complication included mother being transferred [aOR (95%CI) = 2.1 (1.8–2.6)], “high obstetric-risk” [aOR (95%CI) = 1.6 (1.3–1.96)], preeclampsia [aOR (95%CI) = 1.5 (1.2–1.8), mother’s age [aOR (95%CI) = −2% (−3%, −0.1%)] and gestational age [aOR (95%CI) = −8% (−13%, −6%)] increment. This study identified amendable factors associated with peripartum complications in rural referral health settings. Strengthening hospitals’ performance assurance is critical to address the identified factors and improve peripartum outcomes for mothers-neonate dyads.

## 1. Introduction

Africa accounts for 23% of the world’s yearly deliveries, which corresponds to about 32 million births [1]. The previous Millennium Development Goals, which ended in the year 2015, substantially projected health facilities deliveries on the African continent to reach about 50% coverage [2]. Deliveries at health facilities still have insufficient coverage, its improvement remains a core goal in the ongoing Sustainable Development Goal era, so that better maternal and child health outcomes are achieved [3]. Health facility births require high performing quality emergency obstetric care for women around the time of birth. Indeed quality peripartum health care is a well stablished strategy to decrease pregnancy-related maternal and neonatal morbidity and mortality [4,5]. Institutional births provide the opportunity for timely provision of preventative and curative life-saving health interventions to mother and newborn pairs [6,7].

In Mozambique, similarly to other African countries, a significant share of births occurs at district-rural health settings. Maternity health care deserves attention in Mozambique since it presents several opportunities for impactful health interventions [8]. Delays in receiving proper maternity health care are still experienced in Mozambique, including the third delay, that is, a delay in receiving proper health care by the time a woman arrives to hospitals [9,10]. It is no surprise that institutional maternal and perinatal mortality still is high, being mostly caused by manageable causes such as haemorrhage, prolonged labour and eclampsia; furthermore, uterus rupture is highly frequent, despite being an unacceptable cause of maternal neonatal morbimortality in 21st century [11].

Maternity health care studies in Mozambique are limited in their geographical and timing scope. Studies describing women’s peripartum profile, complications, outcomes and associated factors are even more limited [12]. However, young and disempowered women certainly suffer the highest burden of poor peripartum outcomes. It is nevertheless reported that Mozambique has made important progress in emergency obstetric and neonatal care in recent decades [13,14]. It is projected that if Mozambique increases the coverage of life-saving intervention for maternal and neonatal survival, the return in additional maternal–child lives saved will be significant [15,16]. The decrease in maternal and neonatal mortality was significant between 1990 and 2000 in Mozambique, but maternal and neonatal mortality plateaued, respectively, at 452/100.000 and 27/1000 live births over last decade [17,18].

In Mozambique, risk factors for stillbirths, a marker of foetal intrauterine and/or peripartum complications, were described by studies performed in *Tete* and *Zambézia* provinces, which revealed significant opportunities to tackle health-service factors associated with poor peripartum outcomes [19,20]. Furthermore, delays in accessing health care could be managed with assistance from the community health care program which has been active in Mozambique since 2010; however, community health program is currently unprepared to address specific obstetrics and neonatal health needs [21,22]. This contributes to factors of maternal-neonatal adverse outcomes in Mozambique being partially known [23]. On the other hand, an increase in caesarean deliveries is reported, which is *per se* currently an important research topic; access to caesarean sections is reported as being enhanced for the wealthiest and urban women [24] and the underlying factors for women undergoing their first ever caesarean, facing obstetric complications and adverse neonatal outcomes are yet to be described in rural Mozambique.

This study aimed to profile women admitted for childbirth at three referral southern Mozambique rural-district hospitals, across 5 years, and to identify factors associated with first-time caesarean sections and peripartum complications. Although this characterization is limited in its geographical scope, it may be amongst few studies on the matter conducted at referral rural-district health settings in Mozambique. Understanding determinants of caesarean and adverse peripartum outcomes is important to inform the maternal and child health program practices and policies.

## 2. Materials and Methods

### 2.1. Setting

Mozambique is a south-eastern African country with about 30 million inhabitants [17], neighbouring the Republic of South Africa to the South, Tanzania to the North, Malawi, Zimbabwe, Eswatini to the West and the Indian ocean, that is, the Mozambique channel to the East. Data for this study was collected at three southern region district-rural hospitals, namely at *Chicuque* Rural Hospital in *Inhambane* province, *Chokwe* Rural Hospital in *Gaza* province and *Manhiça* District Hospital in *Maputo* province. *Chicuque* Hospital is located approximately 500 km to the North of Mozambique’s capital city *Maputo*, *Chokwe* is approximately 200 km and *Manhiça* is approximately 100 km from Maputo.

According to 2017 Mozambique’s population census and its projections, *Maxixe*—the district where Chicuque hospital is located, in *Inhambane* province—has a population of 129,993 inhabitants, while the *Chokwe* district has 217,019 inhabitants and *Manhiça* has 205,053 inhabitants [17]. Table 1 below describes the monthly averages of maternity health care indicators for the three hospitals. In summary, the routine information system data indicate that all three hospitals record about 200 deliveries monthly. The proportion of HIV-positive women in labour is high, ranging from 13% to 28%; dystocia and caesarean deliveries are substantial, above 8%, and *Chokwe* and *Chicuque* hospitals have much higher proportions of dystocia and caesarean than *Manhiça*, reaching as high as 28%. The direct obstetric complications rate is also high, at a range of 17–94 complications/1000 live births, as opposed to *Manhiça*. The institutional maternal mortality is above 100 deaths/100,000 live births and tracers of neonatal adverse outcome are also high, especially peripartum asphyxia (18–33/1000 live births) [25]. In Mozambique, a significant share of births occurs at district-rural health settings. Indeed, the vast majority (66%) of the Mozambican population reside in rural areas, whilst the fertility rate remains high at about five children per woman’s life cycle, and the natality rate is currently high at 38 births per 1000 population [17,26]. Mozambique is one of the lowest ranking countries on the human development index.

### 2.2. Data Source

Data for this study were retrospectively extracted from clinical files by means of a criteria-based clinical audit, as per the approach described elsewhere [27]. Clinical files include the following data dimensions: (i) demographics such as age and residence; (ii) antenatal information such as parameters for determining gestational age, previous gestations, and obstetric-risk assessment; (iii) specific partograph parameters such as the mother’s vital signs, foetal heartbeat, and delivery dynamics overtime; (iv) medical exam results, prophylaxis, treatments and the medical interventions provided; (v) diagnosis and complications; and (vi) mother and neonatal outcomes.

As per the norm, clinical files and the maternity ward’s logbooks were prospectively filled in by the obstetric clinician practitioner (OCP) attending a delivery. Frontline OCP are midwifes and obstetrics nurses or maternal and neonatal health nurses. For complicated cases, for example, cases requiring caesarean section or other obstetric-related surgery, midwifes count on obstetrics nurses, surgery technicians, doctors or other specialized personnel to intervene [28]. Interventions, medication and clinical reasoning are recorded in semi-structured forms, while the partograph components of clinical files provide the main parameters that need to be recorded in structured fields.

We designed and used a structured data extraction form to aid the audit with a focus on fields essential to understanding the clinical and obstetric presentation of labouring women, and the health care provided. The form was developed with reference to the national perinatal health care guidelines [29] in conjunction with a panel of obstetric nurses, midwifes and doctors, with members invited to the panel being experienced in OCP with rural maternity services for at least a year. Twelve midwifes and obstetric nurses were trained and standardized in study procedures, and they extracted data and audited clinical files in pairs of two under the supervision of health-system and policy researchers from Mozambique’s *Instituto Nacional de Saúde*.

### 2.3. Sampling Strategy

In Mozambique, births are classified as deliveries from pregnancies terminated with at least 28 completed gestation weeks, and/or of a foetus weighting at least 500 g [29,30]. Individual clinical files are paper based, which are archived in a designated area of the hospital or maternity ward after women are discharge from maternity. Archives are clustered on a yearly basis, and then for each year they are again clustered monthly. Our study included clinical files available for the period from January 2013 to December 2018.

We divided each monthly cluster of clinical files into two equal parts. For each month, we picked the half cluster of files in accordance with a randomly pre-generated assignment into group 1 or 2; the halves were then consecutively divided in two batches and the first or second batch was picked according to the randomly assigned group 1 or 2; all partographs from the selected quarter-month were included for data extraction. We then excluded, amongst the selected, files not corresponding to Mozambique’s birth definition. A similar sampling approach was previously developed and used by others in audit-based studies [31]. We did not employ sample size calculation beforehand; however, inherent to the strategy, a large quasi-random sample size (up to 25% of the archive) was included for clinical audits.

### 2.4. Variables and Measures

Maternal age was recorded and extracted as a continuous variable (years); residence in terms of neighbourhood names were recoded as an indicative binary variable accounting for a cut-off of 8 km distance to hospital, which took a value of 1 for “remote” residence and a value of 0 otherwise; the definition of remoteness is based on an 8 km cut-off from a given health facility, a benchmark from Mozambique’s Ministry of Health (MoH) policy for community health workers, and outreach programs [32]; admission, delivery and separation (discharge) date and time were extracted as continuous variables and used to compute the time that elapsed between events—admission to delivery, admission to discharge (separation) and delivery to separation. Other continuous variables include number of gestations; number of deliveries; and current pregnancy gestational age (weeks).

Obstetric risk as per the MoH checklist took the value of 1 if such risks occurred, and 0 otherwise; maternal blood pressure, heart rate, respiratory rate, axillar temperature, haemoglobin, cervix dilatation and uterus height were also extracted as continuous variables; amniotic membranes status took the value of 1 if intact or 0 if ruptured; characteristics of the amniotic fluid took the value of 1 if limpid and 0 otherwise and a clinical case classification at admission of “normal”/absence of complications take value 1 (and 0 otherwise); complications included syndromic obstetric or medical entities such as cephalic-pelvic disproportion, haemorrhage, dystocia, pre-eclampsia and other relatively rare syndromic conditions.

Foetal heart rate at admission was recorded and extracted as a continuous variable, as was also performed for birthweight and Apgar index at 5 min after birth. A Low Apgar index is classified as a score of less than seven. Transvaginal cephalic deliveries with no intercurrences were classified as “normal” taking the value 1 or 0 otherwise; we also extracted a specific variable for caesarean section delivery and twin’s delivery, each taking a categorical variable of 1 if they were to occur and 0 otherwise.

Partographs are tools for standard peripartum monitoring overtime, including records and reasoning about essential maternal–foetal clinical parameters. The tool aids timely clinical decision making [33]. Clinical parameters monitored using partographs include foetal heart rate, cervix dilatation, foetal presentation progress through the birth channel, maternal vital signs, amniotic fluid characteristics, and uterine contractions and their dynamics [34,35].

Data extractors were trained to score the intrapartum monitoring recorded on partographs by assessing tracer clinical parameters and applying a discrete scale with values 0 (no monitoring at all), 25, 50, 75 and 100 (complete monitoring). The data extractors’ benchmark for scoring was the national guidelines on peripartum monitoring [34]. Data extractors clinically adjusted scores with clinical reasoning as appropriate, accounting for a woman’s labour stage at admission to maternity ward. It is indeed common, especially in rural settings, for women to be admitted to maternity wards in advanced labour stages, meaning partograph cannot be used; late labour stage admissions imply that most clinical parameters specific to intrapartum monitoring would not have been monitored overtime nor recorded by attending OCP. For labouring women admitted to maternity wards at early or mid-stages, the monitoring of clinical parameters was scored 100 if in line with the national guidelines and if a clear opportunity to monitor the birth process occurred.

To create a usable variable for the intrapartum monitoring of delivery, we computed a mean score comprising the intrapartum monitoring scores for the following tracer parameters, representing foetal well-being dimensions, maternal vital signs, and toco-dynamics, which are as follows: (i) foetal heart rate, (ii) mother blood pressure, (iii) uterine contractions and (iv) cervix dilatation. The composite “intrapartum monitoring” variable is a final mean score categorized as “adequate monitoring” taking the value of 1 if the mean is at least 75 and value of 0 (inadequate monitoring) otherwise. The 75 point cut-off is usually used in programmatic monitoring and evaluations in Mozambique.

We also extracted tracer parameters for the normative prophylaxis dimension. For maternal peripartum prophylaxis, we considered Oxytocin or Misoprostol administration, Vitamin A, and Antiretroviral therapy as clinically indicated. For the newborns, we considered Vitamin K, Tetracycline ophthalmic ointment and Antiretroviral prophylaxis as clinically indicated. The mentioned prophylaxis administration received a score of 1 if provided and 0 otherwise.

Each of the specific syndromic diagnosis factors such as prolonged labour, cephalic-pelvic disproportion, haemorrhage, pre-eclampsia or eclampsia, foetal distress, asphyxia, stillbirth, low birth weight, preterm newborn were extracted and coded 1 if they occurred, or 0 otherwise. Considering that syndromic diagnosis has a long list of rare possibilities, deliveries with the absence of intercurrences were considered as “normal delivery”, which was coded with a value of 1, or 0 otherwise. “Normal newborn” was considered if newborn complications did not occur, which received the value of 1 or 0 otherwise.

Data extractors, being themselves experienced and trained OCPs, used clinical reasoning to fully audit clinical files and then classify missing information (coded 9999) or not applicable information (coded 8888), allowing excluding missing or not applicable categories when scoring variables with 0.

### 2.5. Statistical Analysis

Descriptive statistics were employed to characterize the clinical and demographic characteristics of women which are contingent to clinical files’ variables. Central tendency measures were computed for continuous variables. Categorical variables were described by absolute and relative frequencies. Unadjusted and adjusted logistic regression were used to investigate the association between women’s recorded demographic and clinical characteristics and first-ever caesarean and peripartum complications. The level of statistical significance was α = 5% or if the estimated parameters’ 95% confidence interval did not cross the null value (null = 1). Data curation and analysis were conducted with SPSS version 24.0 for windows [36].

## 3. Results

The study included 5068 obstetric cases from three southern Mozambique rural hospitals between 2013 and 2018. Table 2 below shows the profile of obstetric cases while Supplementary Table A1 and Table A2 in Appendix A provides a detailed profiling of the cases. Obstetric cases from remote residence in relation to hospitals corresponded to 54%, most of which (71%) were admitted and assisted during weekdays and 44% were admitted during night shifts. Twenty one percent of cases were transferred from other health settings for differentiated and advanced-skill attendance at district-rural hospitals.

The mean age of obstetric cases was 25 years (SD = 7 years) and the mean gestational age was 38 weeks (SD = 2 weeks). Nulliparous women represented 36% of the cases and clinically defined high obstetric risk was recorded for 25% of the obstetric cases. Considering admission blood pressure, 17% cases had hypertension (systolic blood pressure above 140 mmHg and/or diastolic blood pressure above 90 mmHg), 5% had an abnormal foetal heart rate (below 110 or above 160 beats per minute) and 33% were admitted with a completely dilated cervix (10 cm diameter). Nineteen percent of cases underwent caesarean delivery, the vast majority (91%) under rachis anaesthesia (spine-regional) and 86% had a lower abdomen suprapubic and arciform (Pfannenstiel-Kerr) incision (see Table A1 and Table A2 in the Appendix A). Sixteen percent of newborns had a low (less than 7) Apgar index at the first minute, 52% were female and 4% were perinatally asphyxiated as per clinical classification by the assisting OCP teams. Newborns with any of the most common risk diagnoses corresponded to 12%, while 24% of newborns were delivered through complicated labour. Of note is also that 1% of women with previous caesarean section experiences delivered vaginally (see Table A1 and Table A2 in the Appendix A).

**Figure A1:**
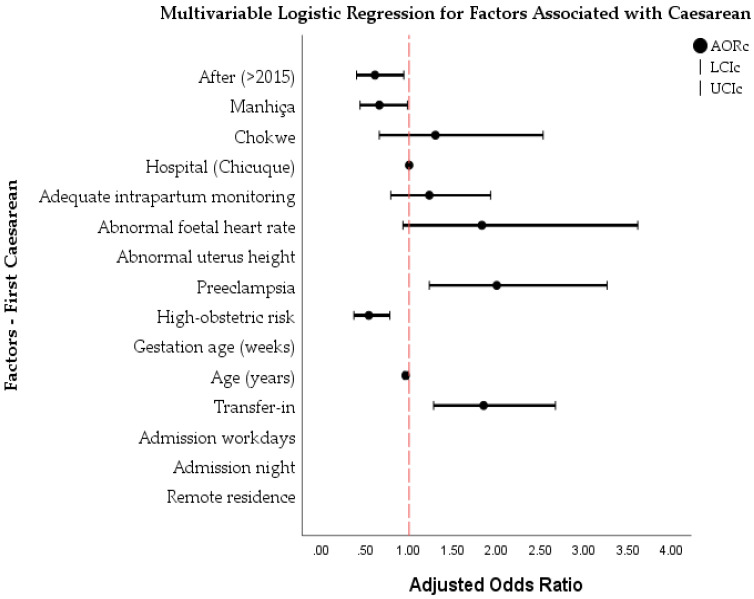
Factors Associated with First Caesarean.

Table 3 below shows, first, the factors associated with first caesarean section, which are also illustrated as Figure A1 in Appendix B. Admissions to maternity by referral from primary health care settings had a 1.8 (95% CI = 1.3-2.6) times increased odds to deliver by caesarean section; cases with preeclampsia (hypertension) had a 2 times (95% CI = 1.2–3.3) increased odds to deliver by caesarean. A lower odds for caesarean delivery was associated with women’s age increment (aOR = 0.96, 95% CI = 0.93–0.99), deliveries at *Manhiça* hospital as opposed to *Chicuque* (aOR = 0.66, 95% CI=0.44-0.98), high obstetric-risk (aOR = 0.54, 95% CI = 0.37–0.78) and assistance being after 2015 as opposed to before (aOR = 0.61, 95% CI = 0.39–0.94).

Second, as shown in Table 3, an increased odds of obstetric complication was associated with admission by referral (aOR = 3.4, 95% CI = 2.8–4.1), high obstetric-risk as per antenatal care classification (aOR = 4.7, 95% CI = 3.9–5.7), cases at *Manhiça* as opposed to *Chicuque* (aOR = 3.3, 95% CI = 2.6–4.0) and deliveries after 2015 (aOR = 1.5, 95% CI = 1.2–1.8). On the other hand, a lower odds of obstetric complication was associated with admissions on night shifts (−28.3%, 95% CI = −40.9%, −13.1%) and deliveries in *Chokwe* (−51.9%, 95% CI = −74.6%, −38.1%) as opposed to *Chicuque* hospital.

Third, Table 3 also shows factors associated with neonatal complications. Cases admitted by referral from primary health care services had a 2.1 times (95% CI = 1.8–2.6) higher odds of neonatal complication; cases with high obstetric-risk had a 1.6 (95% CI = 1.3–1.96) odds of neonatal complications, preeclampsia (hypertension) at admission implied 1.5 (95% CI = 1.2–1.8) times more complications, and delivering at *Manhiça* was associated with 1.5 (95% CI = 1.2–1.9) times more complications. Lower neonatal complications were associated with admission during night shifts (−24%, 95% CI = −37%, −8%), mother’s age increment (−2%, 95% CI = −3%, −0.1%) and gestational age increment (−8%, 95% CI = −13%, −6%).

## 4. Discussion

This study comprehensively characterized obstetric cases assisted at three key first referral district-rural hospitals in southern Mozambique. To our best knowledge, this is the first study to investigate factors associated with obstetric cases undergoing caesarean delivery amongst women with no prior caesarean section, and factors associated with maternal and neonatal composite adverse outcomes in Mozambique’s district-rural health settings.

The ability to provide comprehensive emergency obstetric and neonatal care is a main desired feature for district-rural hospitals in Mozambique’s health services [8,37,38,39]. As expected, results pointed to relatively complex obstetric cases clustered for clinical assistance at district-rural hospitals. For example, despite the mean gestational age of labouring women being 38 weeks (SD = 2 weeks), the mean age of women was 25 years (SD = 7 years), only 26% were nulliparous, 25% had a clinically defined high obstetric-risk, 17% had hypertensive disorder, 33% were admitted at the advanced labour stage, and about 19% delivered by caesarean section.

The study identified factors associated with delivery by caesarean section, which includes admission type, women’s age, clinically determined high obstetric-risk, and pre-eclampsia as a separate nosology. Several studies from the African region identified a similar profile and factors [40,41,42,43], although these studies did not excluded cases with prior caesarean section as performed in this study. A direct comparison of our findings to the above listed African studies is rather limited because articles reporting composite complications in the same way are generally unavailable; rather, most previously published articles analysed factors associated with specific nosology such as pre-eclampsia, cardiomyopathy, pre-term, occiput posterior presentation, stillbirths and sepsis as outcomes. As for the trend of caesarean sections, they are reported to be increasing in Mozambique, but this is based on studies of population-level data [24] while our study is hospital based.

We noted that cases assisted at *Manhiça* hospital had a lower odds of undergoing caesarean section. It is possible that *Manhiça* hospital refers cases for caesarean section more frequently since this rural hospital is closest to *Maputo* province, a geographical area better serviced in terms of hospital availability, equipment, and human resources for advanced obstetric care. We cannot exclude the disruption in the running of *Manhiça* hospital during the study period as a factor for admitted cases having higher odds of complications, with a lower odds of receiving a caesarean section.

It was also noteworthy that overall, more recent cases (2015 onwards) had a lower odds of undergoing caesarean section. This is a particularly intriguing finding since caesarean delivery was expected to increase in the period post 2015, especially considering the increase in caesareans observed by health survey analysis conducted by Long and colleagues [24]. The context in Mozambique’s hospitals is that a quality intervention—model maternity program—was designed in 2011 and effectively rolled out in 2015 and onward [37]. The program covered all three studied hospitals. It is thereby reasonable to propose that decreasing the occurrence of unnecessary and unsafe caesarean sections may have been an intrinsic model maternity program intervention goal. However, rural hospitals, being the first level referral health settings, do cluster complicated obstetric cases, so a decrease in caesarean sections should not be expected. Indeed, lower odds of undergoing a caesarean after 2015, while the workload at hospitals did not change significantly, is an interesting finding that should be investigated in further research.

This study found factors associated with the obstetric complication occurrence amongst assisted cases to be similar to those associated with a person undergoing their first ever caesarean delivery. The study identified this expected intrinsic correlation; in fact, among admissions to maternity wards by either referral or emergency, high obstetric risk was clinically classified using a national check list of clinical features, including factors associated with both complications and first ever caesarean section. Our findings corroborate the results of several studies from southern Africa on factors associated with peripartum complications, either for the mother or for the neonate’s nosology [44,45,46,47,48,49,50]. This is despite methodological differences between our study and others, in terms of outcomes. For example, other studies mostly focused on foetal outcomes, and some on maternal near-missed outcomes.

An interesting set of associations can be identified by looking at *Manhiça* hospital outputs in Table 3. The results indicated a lower odds for caesarean at *Manhica* but higher odds for complications. This latter divergence between complications and caesarean is suggestive that *Manhiça*, again, being a district relatively close to *Maputo*, more frequently refers complicated cases. Although *Manhiça* has a district hospital, this may be at a more privileged geographical area in terms of hospital availability, which includes hospitals from *Maputo* province and *Maputo* city. *Chicuque* and *Chokwe* hospitals may have to manage obstetric cases complications onsite, with little further referral to provincial hospitals. Further studies and analyses about differences in terms of specific factors across hospitals may be needed to ascertain the roots causes for such diverse odds at *Manhiça* district hospital.

The study found negative associations between night shifts admissions and obstetric complications occurrence. This interesting finding may be related to continuity of care. Night shifts admission implies obstetric cases being assisted at critical labour stages only during the following day shift. Supposedly, there is a continuity of care from night admission to the upcoming morning, and onwards. Teams at hospitals are usually better staffed and well-resourced between 7 am and 4 pm, thus providing better intrapartum monitoring, technical quality, and early complications management. Usually, health-worker training at hospitals, which increases the manpower with finalist students, occurs at daytime as well. We have not found studies exploring health service factors and timing underpinning peripartum outcomes.

Contextual and endogeneity factors were limitedly accounted for in this study, which may explain the lower odds for cases, at *Chokwe* hospital, to register complications as opposed to *Chicuque* hospital. The *Chicuque* hospital referral catchment area is larger than *Chokwe’s*, in terms of distance, the number of primary health facilities for referrals and population remoteness [17,51]. The size of the *Chicuque* catchment area and its remoteness may be the root factor for higher complications clustering in this hospital. Routine data shown in Table 1 corroborate our finding, pointing to *Chicuque* clustering more adverse outcomes.

Foetal and neonatal complications are intrinsically connected to obstetric complications, and thereby connected to caesarean sections [6,7,29]. It is therefore no surprise but worth noting overlapping on the factors associated with the three studied outcomes, the foetal complications, caesarean section, and obstetric complications. The type of admission, whether by referral or emergency, high maternal obstetric risk and maternal hypertensive disorder were consistent factors determining neonatal and obstetric complications, and caesarean section. It was also verified and expected that the closer to term the gestational age is, the lower the odds for neonatal complications. Our study results were corroborated by several other studies from similar contexts [19,52,53,54,55,56], though there are some methodological and outcome definitional differences across studies and between others and our study.

Our results revealed that the majority women (64%) were multiparous although relatively young (mean age 25 years, SD = 7). It is thereby evident that complications odds decreased with increasing age. The context in Mozambique is of high early pregnancies prevalence [57], a known determinant of obstetrics complications and poor maternal and neonatal outcomes [43,58,59]. As a woman’s age increases, better obstetric and neonatal outcomes are thereby expected, even physiologically.

The study had some limitations, but also strengths. Our sampling intrinsically had selection bias since the study was conducted at referral hospitals. The study excluded cases with maternal or neonatal death outcomes—since specific archives were inaccessible. Files corresponding to maternal and neonatal deaths were sent out monthly to maternal and neonatal death audit committees and not kept at hospitals. Nevertheless, the study analysed women’s profiles in general and analysed intercurrences of similar and preceding known causes of maternal and neonatal deaths. We do not believe that normal or complicated case records were missed. Not finding files in archives should be rare besides for cases that end in deaths, since monthly archiving is a long-term standard and practice at Mozambican hospitals. Moreover, we trust our findings inform clearly on peripartum health care practices in the rural health setting context. The study covered principal or essential parameters and indicators used for policy making and health program discussions.

Measurement bias is also a possible study limitation since data extraction was performed with administrative documents and clinical files, which are mostly designed for clinical practice rather than research. Data were retrospectively extracted. Missing information was also introduced by variations in clinical reasoning and by differences in completeness of clinical forms used by assisting clinicians. In the study data extraction process, we were not able to fully control for precision and recording quality during retrospective clinical practice. However, the study focused on essential and mandatory variables in clinical practice, which are subject to continuous supervision, and of interest for quality improvement programs [8,29,37]. The sample was also designed to be large. Incomplete information and not applicable situation were carefully coded during the data extraction process, thereby controlling for missing or not applicable information during the analysis.

Furthermore, the measurement bias in this study applies to the entire study period and all included hospitals, which minimizes its impact. Obstetric clinical management and practices in Mozambique are based on national guidelines, continuous supervisions, quality checks and quality improvement programs, and are also nationally guided [29,34,60]. Thus, policy, guidance, and interventions effects should in theory be similar across hospitals, thereby minimizing measurements bias. Measurement bias could in addition result from the intrapartum monitoring scoring that was applied. We relied on OCP-data-extractors’ clinical reasoning to adjust the intrapartum monitoring scores which is prone to subjectivity; however, the study counted on experienced OCPs in terms of knowledge and practice of national guidelines, who benefited from standardization on study procedures and were continuously supervised and supported for quality data extraction.

The consistency of findings about factors associated with complications and caesarean sections is a major study strength. This study also adds value to the utilization of a considerable volume of administrative data despite the above-mentioned limitations.

## 5. Conclusions

Obstetric cases assisted at district-rural hospitals in southern Mozambique characteristically include younger women, are multiparous, and mostly include term pregnancy. The hospitals cluster complicated cases. Women’s age, clinically determined obstetric risk at antenatal consultation, being referred-in during labour and hypertensive disorder, as expected, were confirmed as factors associated with peripartum complications. The outbound referral of complicated cases to provincial hospitals may be a common practice at a few, but not all, hospitals.

An interesting finding to be investigated by further studies is that in the period after 2015, a higher odds of obstetric complication was recorded amongst cases, but a lower caesarean delivery odds was also found. The period after 2015 corresponds to the full rollout and routinization of quality and performance improvement interventions. This finding is divergent from average indicators based on the routine health information system. It is desirable that quality improvement leads to better complication screening but also a more precise caesarean section prescription. If the latter is the case, then we found a very positive effect in Mozambique in terms of quality and performance improvement initiatives. The results suggest that the quality health care improvement interventions and rural hospitals readiness remain a top priority in Mozambique.

## Figures and Tables

**Table 1:** Maternity health care indicators (2016–2018), *Chicuque*, *Chokwe*, *Manhiça* hospitals, Southern Mozambique.

	Hospital		
Indicators: Monthly Averages	Manhiça	Chokwe	Chicuque
Completed Partograph, %	90%	95%	76%
Deliveries All Forms, n	213	208	198
HIV Positive, %	28%	24%	13%
Live Newborns, n	211	202	195
Caesarean, %	12%	19%	25%
Dystocia Delivery, %	12%	21%	28%
Peripartum Haemorrhage, n/1000 LB	8	22	39
Low Birth Weight, n/1000 LB	53	50	87
Pre-Term, n/1000 LB	22	24	36
Asphyxia, n/1000 LB	33	18	33
Direct Obstetric Complication, n/1000 LB	54	108	266
All Obstetric Complication, n/1000 LB	90	116	287
Institutional MMR, n/100,000LB	100	106	228

LB = live births; MMR = maternal mortality ratio. Source: health information system, Ministry of Health [25].

**Table 2:** Selected sample characteristics, *Chicuque*, *Chokwe* and *Manhiça* hospitals 2013–2018.

Variables	Estimates **	n
Remote residence, % [95% CI]	54 [52–55]	5059
Admission workdays, % [95% CI]	71 [70–72]	5068
Transfer-in, % [95% CI]	21 [20–22]	5034
Age(years), mean, [median, IQR]	25 [24,11]	5068
Gestation age (weeks), mean [median, IQR]	38 [39,3]	5068
Nulliparous, % [95% CI]	36 [35–38]	5064
High-obstetric risk, % [95% CI]	25 [25–26]	5048
Preeclampsia (hypertension), % [95% CI]	17 [16–18]	4784
Abnormal foetal heart rate, % [95% CI]	5 [5–6]	4671
Cervix completely dilated, % [95% CI]	33 [31–35]	2526
Prior caesarean, % [95% CI]	5 [4–6]	5068
Normal labour, % [95% CI]	76 [75–77]	5068
Caesarean cephalic pelvis disproportion, % [95% CI]	27 [24–30]	941
Caesarean antepartum haemorrhage, % [95% CI]	9 [7–11]	941
Caesarean delivery (all causes), % [95% CI]	19 [18–20]	5066
Caesarean, vacuum extraction, podalic, % [95% CI]	20 [19–21]	4999
Newborn at risk (all diagnosis), % [95% CI]	12 [11–13]	4973
Delivery with any complication, % [95% CI]	24 [22–25]	5019
Time admission-delivery (h:m), median, [IQR] ^a^	1:39, [4: 30]	3556
Time admission-discharge (days), median, [IQR]	2, [1]	4947

** rounded % for readability; CI (confidence interval); IQR (interquartile range); ^a^ analysis restricted to deliveries within 24 h.

**Table 3:** Factors associated with caesarean, maternal and neonatal complications.

	OR [95% CI]	*p*	aOR [95% CI]	*p*
**Caesarean amongst cases without previous caesarean**				
Remote residence	0.82 [0.59–1.15]	0.253		
Admission night	1.01 [0.73–1.4]	0.935		
Admission workdays	0.77 [0.54–1.1]	0.152		
Transfer-in	2.08 [1.51–2.87]	**0.000**	1.85 [1.28–2.67]	**0.001**
Age (years)	0.95 [0.93–0.97]	**0.000**	0.96 [0.94–0.99]	**0.004**
Gestation age (weeks)	1.0 [0.94–1.07]	0.951		
High-obstetric risk	0.57 [0.42–0.78]	**0.000**	0.54 [0.37–0.78]	0.001
Preeclampsia	2.32 [1.5–3.59]	**0.000**	2.0 [1.23–3.26]	**0.005**
Abnormal foetal heart rate	2.11 [1.18–3.79]	**0.012**	1.83 [0.93–3.61]	0.080
Abnormal uterus height	1.11 [0.75–1.65]	0.608		
Adequate intrapartum monitoring	1.34 [0.92–1.98]	0.132	1.23 [0.79–1.93]	0.357
Hospital (Chicuque)	Ref			
Chokwe	1.01 [0.65–1.59]	0.950	1.3 [0.66–2.53]	0.451
Manhiça	0.54 [0.38–0.76]	**0.000**	0.66 [0.44–0.98]	**0.041**
After (>2015)	0.59 [0.4–0.86]	**0.006**	0.61 [0.4–0.94]	**0.026**
Obstetrics complication **				
Remote residence	1.81 [1.55–2.12]	**0.000**	1.03 [0.85–1.25]	0.777
Admission night	0.62 [0.53–0.72]	**0.000**	0.72 [0.59–0.87]	**0.001**
Admission workdays	1.02 [0.87–1.21]	0.786		
Transfer-in	5.1 [4.34–6]	**0.000**	3.39 [2.78–4.14]	0.000
Age (years)	1.0 [0.99–1.01]	0.738		
Gestation age (weeks)	1.0 [0.97–1.03]	0.772		
High-obstetric risk	5.31 [4.53–6.23]	**0.000**	4.69 [3.86–5.69]	**0.000**
Adequate intrapartum monitoring	0.59 [0.5–0.71]	**0.000**	0.82 [0.67–1.01]	0.064
Hospital (Chicuque)	Ref	**0.000**		
Chokwe	0.53 [0.44–0.64]	**0.000**	0.48 [0.36–0.63]	**0.000**
Manhiça	2.33 [1.94–2.78]	**0.000**	3.25 [2.61–4.05]	**0.000**
After (>2015)	2.07 [1.74–2.46]	**0.000**	1.48 [1.21–1.83]	**0.000**
**Neonatal complication**				
Remote residence	1.47 [1.25–1.74]	**0.000**	1.12 [0.92–1.35]	0.251
Admission night	0.72 [0.61–0.85]	**0.000**	0.76 [0.63–0.92]	**0.005**
Admission workdays	0.86 [0.73–1.02]	0.091		
Transfer-in	2.79 [2.35–3.31]	**0.000**	2.15 [1.75–2.63]	**0.000**
Age (years)	0.99 [0.97–1]	**0.016**	0.98 [0.97–1]	**0.009**
Gestation age (weeks)	0.9 [0.88–0.93]	**0.000**	0.91 [0.87–0.94]	**0.000**
High-obstetric risk	2.12 [1.79–2.51]	**0.000**	1.6 [1.31–1.96]	**0.000**
Preeclampsia	1.83 [1.51–2.22]	**0.000**	1.46 [1.18–1.81]	**0.001**
Abnormal uterus height	0.88 [0.68–1.15]	0.348		
Adequate intrapartum monitoring	0.83 [0.7–0.99]	0.043	0.94 [0.77–1.14]	0.541
Hospital (Chicuque)	Ref			
Chokwe	0.74 [0.62–0.89]	**0.001**	0.91 [0.72–1.14]	0.419
Manhiça	1.28 [1.04–1.57]	**0.019**	1.48 [1.17–1.86]	**0.001**
After (>2015)	1.22 [1.03–1.44]	**0.024**	1.12 [0.93–1.37]	0.237

** “Preeclampsia” and “abnormal uterus height” are part of dependent variable “obstetrics complication”; OR (Odds Ratio); aOR (Adjusted Odds Ratio); CI (Confidence Interval); Bold *p* =statistically significant probability.

**Figure A2:**
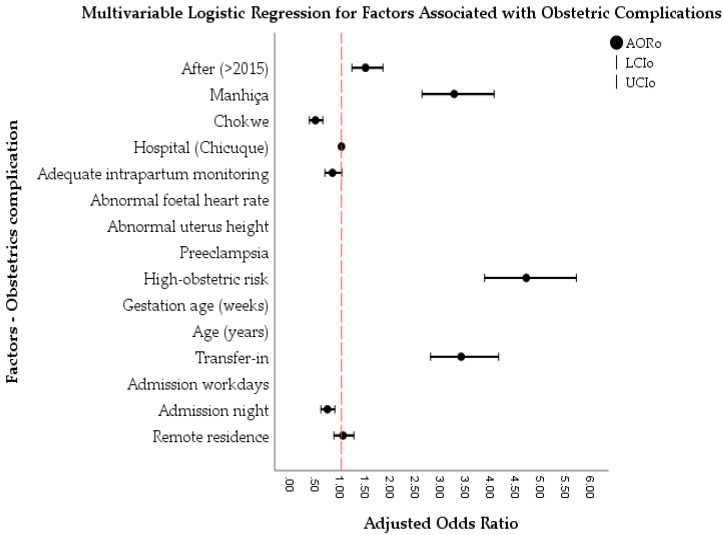
Factors Associated with Obstetric Complications.

**Figure A3:**
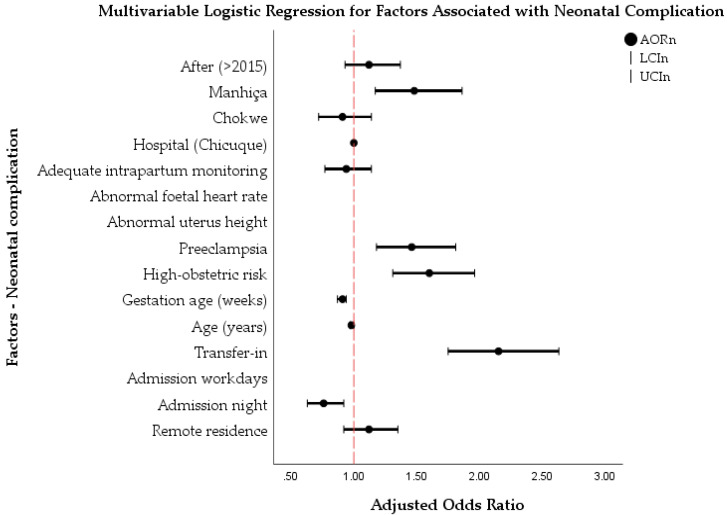
Factors Associated with Neonatal Complications.

## Data Availability

The data presented in this study are available on reasonable request from the corresponding author.

## Appendix A

**Table A1:** Obstetric cases extended demographic and clinical profile, *Chicuque*, *Chokwe* and *Manhiça* hospitals, Southern Mozambique 2013–2018.

Dimensions	Variables	Estimate	95% CI		n	Median	IQR
General info. admission	Remote residence	54.0%	52.3%	55.1%	5059		
	Admission workdays	71.0%	69.7%	72.2%	5068		
	Admission night	44.0%	42.8%	45.5%	5068		
	Transfer-in	21.0%	19.6%	21.9%	5034		
	Age(years)	25	25	25	5068	24	11
Clinical admission	Gestation age (weeks)	38	38	39	5068	39	3
	Nuliparous	36.0%	34.6%	34.7%	5064		
	High-obstetric risk	25.0%	24.6%	25.8%	5048		
	PreEclampsia (hypertension)	17.0%	15.9%	18.1%	4784		
	Abnormal respiratory rate	3.0%	1.6%	3.9%	833		
	Abnormal heart rate	24.0%	22.9%	25.6%	3957		
	Abnormal foetal heart rate	5.0%	4.7%	6.0%	4671		
	Cervix completely dilated	33.0%	31.3%	35.0%	2526		
Delivery	Delivery by caesarean	19.0%	17.5%	19.6%	5066		
	Rachianaesthesia	91.0%	89.4%	93.2%	859		
	Pfannestiel incision	86.0%	83.7%	88.5%	805		
Diagnosis caesarean	Caesarean previous caesarian	33.0%	29.7%	35.7%	941		
	Caesarean cefalo-pelvic disproportion	27.0%	24.2%	29.8%	941		
	Caesarean foetal asphyxia	28.0%	24.9%	30.6%	941		
	Caesarean haemorrhage	9.0%	6.9%	10.5%	941		
	Caesarean prolonged labour NE	9.0%	6.8%	10.4%	941		
	Caesarean other dystocia NE	7.0%	5.8%	9.1%	941		
	Caesarean Eclampsia	5.0%	3.8%	6.7%	937		

**Table A2:** Obstetric cases extended demographic and clinical profile, Chicuque, Chokwe and Manhiça hospitals, Southern Mozambique 2013–2018.

Dimensions	Variables	Estimate	CILow	CIUpper	n	Median	IQR
Neonate	Severe low Apgar (<7)	16.0%	13.5%	18.1%	937		
	Newborn female	52.0%	50.7%	53.5%	5009		
	Low birth weight (<2.5 kg)	14.0%	13.2%	15.2%	4839		
Anaemia	Women moderate or severe anaemia	27.0%	24.5%	30.3%	934		
Newborn Discharge Diagnosis	Missing diagnosis	1.0%	1.1%	1.7%	5068		
	Macrossomic	0.0%	0.1%	0.3%	5068		
	Neonatal death	0.0%	0.3%	0.7%	5068		
	Asphyxia 1	4.0%	3.6%	4.7%	5068		
	Meconium aspiration	1.0%	0.5%	1.0%	5068		
	Stillbirth	4.0%	3.1%	4.2%	5068		
	Normal	86.0%	85.5%	87.4%	5068		
	Pre-term	1.0%	1.1%	1.8%	5068		
	Sepsis	0.0%	0.0%	0.1%	5068		
	Asphyxia 2 (Intrauterine distress)	0.4%	0.3%	0.6%	5068		
	Y weight	1.0%	0.6%	1.1%	5068		
Neonate complication	Newborn at risk (any diagnosis)	12.0%	11.3%	13.1%	4973		
	Newborn: not well	25.0%	22.7%	26.6%	4973		
Women complication	Mother: any obstetric complication	16.0%	15.1%	17.2%	5047		
	Delivery with complication	24.0%	22.4%	24.7%	5019		
	Vaginal delivery previous caesarean	1.0%	0.6%	1.1%	5068		
Time to events	Time to deliveries (h:m), mean	3:09	3:01	3:17	3556	1:39	4:30
	Time to discharge (days)	3	3	3	4947	2	1

## Appendix C

Authors list and affiliation for the Group “Performance in Obstetrics and Evaluation of Maternities” (POEM-group).

Claudio Muianga ^a^, Yasser Gulamo ^a^, Ofélia Rambique ^b^, Carlos Botão ^b^, Gildo Muchanga ^b,^^†^, Euridsse Amade ^b^, Laurentino Cumbi ^b^, Nurbai Calú ^a^, Cidália Baloi ^a^ and Janeth Dulá ^b^, Sérgio Chicumbe ^b^

a. World Health Organization representation in Mozambique, Rua Joseph Kizerbo, 227, P.O. Box 377, Maputo, Mozambique; muiangac@who.int (C.M.); gulamoy@who.int (Y.G.); calun@who.int (N.C.); baloic@who.int (C.B.)
b. Health System and Policy Program, Instituto Nacional de Saúde, Estrada Nacional 1, Vila de Marracuene, Parcela 3943, Província de Maputo, Mozambique; sergio.chicumbe@ins.gov.mz (S.C.); dula.janeth@gmail.com (J.D.); laurentino.cumbi@ins.gov.mz (L.C.); euridsse.amade@ins.gov.mz (E.A.); carlos.botao@ins.gov.mz (C.B.); ofelia.rambique@gmail.com (O.R.)

† *in memoriam*
